# Neonatal Hepatitis as First Manifestation of Hyperimmunoglobulinemia D Syndrome

**DOI:** 10.1155/2014/936890

**Published:** 2014-03-03

**Authors:** Marie-Louise von Linstow, Vibeke Rosenfeldt

**Affiliations:** Department of Paediatrics, Hvidovre University Hospital, Kettegaard Alle 30, 2650 Hvidovre, Denmark

## Abstract

Hyper IgD syndrome (HIDS) is a rare metabolic autoinflammatory syndrome characterised by recurrent febrile episodes, accompanied by various inflammatory symptoms. We present a case of severe HIDS in a young girl, whose symptoms started in the neonatal period with hepatomegaly, hepatitis, thrombocytopenia, and conjugated hyperbilirubinemia. From the age of five months, the child had recurrent febrile episodes, stomatitis, adenitis, and persistent hepatomegaly. The diagnosis of HIDS was established when she was three years and eight months old. This case report suggests that HIDS should be included in the differential diagnosis of neonatal hepatitis and conjugated hyperbilirubinemia.

## 1. Background

Hyper IgD syndrome (HIDS) is a rare metabolic autoinflammatory syndrome characterised by recurrent febrile episodes, accompanied by lymphadenopathy, exanthema, joint pain, abdominal pain, diarrhoea, vomiting, headache, hepatomegaly, myalgia, conjunctivitis, and oral ulcers [1, 2].

HIDS is also termed mevalonate kinase deficiency, as mutations in the mevalonate kinase (MVK) gene are responsible for the autosomal recessive pattern of inheritance seen in HIDS [3, 4]. MVK deficiency is present both in HIDS and in the more severe phenotype mevalonate aciduria (MA). MA is characterized by recurrent febrile crises, severe mental retardation, ataxia, failure to thrive, cataracts, and dysmorphic features. These children often die in early childhood [5].

Only two cases of HIDS, both from Finland, have been described in Scandinavian children [6]. We here present a case of HIDS in a girl, in whom typical symptoms were registered from the age of five months. However, severe hepatitis and thrombocytopenia in the neonatal period might have been the first manifestation of HIDS.

## 2. Case Presentation

The girl was born in Tórshavn, Faeroe Islands. The mother was a primipara and previously healthy with an uneventful pregnancy. The parents were nonconsanguineous, the mother being of Columbian and the father of Faroese ancestry. Spontaneous labour occurred in gestational age 38 weeks. Due to slow progress, green amniotic fluid, polyhydramnion, and a pathological cardiotocography, an acute caesarean section was performed. Apgar scores were 3, 6, and 9 in 1, 5, and 10 minutes, respectively. Birth weight was 3,510 g. K-vitamin was administered. The mother remained afebrile during and after delivery.

The child had a normal physical examination, except from a petechial rash. She was suspected for sepsis and started on ampicillin and gentamycin. Abdominal ultrasound (US) showed an enlarged liver with a normal structure. Within the first 24 hours, she became icteric and was transferred to specialised neonatal care in Copenhagen.

Treatment was initiated with nasal-continuous-positive-airway-pressure, phototherapy, antibiotics, diuretics, and blood transfusions. She was anaemic, with a decreased platelet count, elevated liver enzymes, elevated leucocyte count, and a very high C-reactive protein (CRP). Blood culture was negative. A computed tomography (CT) scan of the thorax and abdomen confirmed a severely enlarged homogeneous liver.  Table 1 lists the investigations performed in the neonatal period. Laboratory values gradually normalised and the hepatomegaly decreased. No infectious or metabolic aetiology of hepatitis was identified. The conclusion for her neonatal symptoms was assumed to be a congenital viral infection without known aetiology.

Three years old, she was admitted to the outpatient clinic because of a CRP level of 138 mg/L and haemoglobin of 5.3 mmol/L measured by the general practitioner.

Since the age of three months, she had suffered from recurrent white coatings and blisters on the tongue. Moreover, from five months of age, after vaccination with the DTaP/IPV (polio)/Hib vaccine, she has had episodes of fever 39-40°C every second week, accompanied by a cervical lymphadenitis, rash, and more blisters in the mouth. Each illness episode lasted between 6 and 14 days. An adenotonsillectomy seven months prior to admission had no relief on the symptoms. Growth and development were normal.

Physical examination was normal, except from pallor due to anaemia. Haemoglobin was 5.8 mmol/L, leucocytes 12.7 × 10^9^/L, thrombocytes 505 × 10^9^/L, Erythrocyte sedimentation rate (ESR) 29 mm/h, and CRP 7 mg/L, and a throat swab was negative. On the day of first examination, the patient was afebrile. Human immunodeficiency virus (HIV), cytomegalovirus (CMV), and Epstein-Barr virus (EBV) antibodies were negative. The girl was sent home with information of going directly to the Paediatric Emergency Department in case of fever.

A month later she was admitted with fever 38.5°C and rhinitis. She had tender cervical adenitis, but otherwise a normal examination. A chest X-ray was normal. CRP was 142 mg/L and ESR 72 mm/h. She was followed in the outpatient clinic and a spontaneous drop in CRP to 72 mg/L was observed and the fever resolved. At this point, further investigations were performed, including a normal fractionated haemoglobin (Table 2). IgA was elevated, and IgD was initially normal.

Two weeks later, she was admitted with rhinitis, fever 38°C, cervical adenitis, and painful stomatitis. CRP was 145 mg/L, ESR 76 mm/h, and leukocytes 23 × 10^9^/L. She was treated empirically with Amoxicillin/clavulanic acid for 10 days and sent home.

Three weeks later, she was admitted with fever and a rash and a CRP of 104 mg/L. Physical examination revealed a macular rash on the legs and no petechiae. She was discharged without treatment.

Five days later, she returned with fever and persistent rash. The temperature was 39.3°C. On examination, she was alert and oriented. Lymphadenitis colli and a nonspecific blanching exanthema were present. CRP was 235 mg/L, urine dip-stick was normal, and chest X-ray was without infiltrates. She was treated with cefuroxime, gentamycin, and Amoxicillin/clavulanic acid. The rash disappeared, and no peeling was observed. During her hospitalisation, a range of investigations was performed (Table 3).

Final test results were ready four weeks later, and a remarkable high level of IgD of 2012 IU/mL was noted. A diagnosis of HIDS was confirmed by increased excretion of mevalonic acid in the urine of 21.8 *μ*mol/mmol and by DNA diagnostics showing the patient to be compound heterozygote for the V377I and the c.417insC mutations in the MVK gene.

Until the age of six years, laboratory tests in between fever attacks showed signs of chronic inflammation with anaemia (6.1–6.5 mmol/L), moderately elevated ESR (18–35 mm/h), and thrombocytosis. Abdominal US scan, performed twice in 2008 and 2010, showed slight enlargement of the liver. In 2011, she had approximately 100 sick days. Steroids administered immediately after the first symptoms only had an effect on the fever, but not on arthralgias, rash, oral ulcers, or abdominal pain. In 2012, she had 90 sick days, and in April 2013 it was decided to start her on etanercept. After increasing the dose from 15 mg to 25 mg subcutaneously twice weekly, attacks are shorter (3-4 days), now occurring every second month without accompanying arthralgias or abdominal pain.

## 3. Discussion

This case report illustrates that HIDS is a devastating disease, significantly affecting the quality of life. Our patient had severe symptoms of hepatomegaly, hepatitis, and conjugated hyperbilirubinemia in the neonatal period, and typical symptoms of HIDS started at the age of five months. In her first years of life, clinical and laboratory signs of inflammation were continuously present, even in between fever attacks, which have not previously been reported in the literature as a typical feature of HIDS. Hepatitis with conjugated hyperbilirubinemia and low platelet count in the neonatal period could have been caused by the severe systemic inflammation, and indeed signs of low level chronic inflammation and moderate hepatomegaly were still present at the age of three years. Unfortunately, a liver biopsy was never performed, making us unable to describe the histological characteristics of the inflammation.

Few cases of children with HIDS presenting with hepatomegaly and hepatitis have been described. A Japanese girl presented with neonatal-onset chronic hepatitis but lacked the typical HIDS features until the age of 32 months [7]. Another child developed liver disease at six months of age and was diagnosed with HIDS at 24 months of age [8]. The spectrum of symptoms seen in our patient, such as conjugated hyperbilirubinemia, thrombocytopenia, petechiae, hepatomegaly, hepatitis, and anaemia, has previously been described as the first manifestation of the severe disease mevalonate aciduria in newborns [9–11]. As HIDS and MA represent two phenotypic ends of the spectrum of MVK deficiency, it is reasonable to assume that identical symptoms can present at birth and that the neonatal symptoms in our patient were a manifestation of HIDS.

This case report emphasizes that an initial normal IgD level cannot rule out severe HIDS. Furthermore, it illustrates the importance of including HIDS in the differential diagnosis of neonatal hepatitis and conjugated hyperbilirubinemia.

## Figures and Tables

**Table 1:** Investigations performed in the neonatal period.

Examination	Test result
Haemoglobin (range)	5.0–9.5 mmol/L
Leucocytes (highest)	45 × 10^9^/L
Thrombocytes (lowest)	17 × 10^9^/L
CRP (highest)	110 mg/L
Bilirubin total (highest)	552 µmol/L
Bilirubin conjugated (highest)	392 µmol/L
ALAT (highest)	281 U/L
Alkaline phosphatase (highest)	483 U/L
Lactate dehydrogenase	3235 U/L
Urate	0.38 mmol/L
Lactate	2.2 mmol/L
Blood culture	Negative
Blood smear	Extramedullary haematopoiesis no lymphoblasts
Bone marrow aspiration	No malignancy
CMV serology	Negative
CMV PCR in blood and urine	Negative
EBV serology	Negative
Toxoplasmosis serology	Negative
VZV serology	Negative
Urine metabolic screen	Normal
X-ray thorax	No infiltrates
X-ray abdomen	Normal
US abdomen	Marked hepatomegaly
CT thorax and abdomen	Marked hepatomegaly
US cerebrum	2.2 × 2.5 cm echogenic process
CT cerebrum	Haemorragia in posterior fossa
Electrocardiogram	Normal
Echocardiography	Normal
Lumbar puncture	Unsuccessful

**Table 2:** Investigations performed in the outpatient clinic and at first acute hospitalisation in Denmark 3.5 years of age.

Investigation	Result
Haemoglobin	5.2 mmol/L
Leucocytes	10.9 × 10^9^/L
Thrombocytes	505 × 10^9^/L
CRP	142 mg/L
Erythrocyte sedimentation rate	72 mm/h
Iron	2 µmol/L (ref.: 5–20)
Haptoglobin	4.41 g/L (ref.: 0.35–1.85)
Transferrin	34 µmol/L (ref.: 24–41)
Urate	0.49 mmol/L (ref.: 0.15–0.35)
Lactate dehydrogenase	203 U/L (ref.: 155–450)
IgD	100 IU/mL
IgG	12.3 g/L (ref.: 3.4–9.1)
IgA	3.08 g/L (ref.: 0.12–1.49)
IgM	1.64 g/L (ref.: 0.39–2.08)
IgG subclasses	Normal
Blood culture	Negative
Blood smear	Reactive, no lymphoblasts
HIV antibodies	Negative
Complement defect screening	Normal
Vaccination response to HiB	1.41 µg/mL (ref.: >1 µg/mL)
Vaccination response to diphtheria	1.6 IE/mL (ref: >0.01 IE/mL)
Vaccination response to tetanus	0.98 IE/mL (ref: >0.1 IE/mL)
Mannan binding lectin (MBL)	220 µg/L
Haemoglobin electrophoresis	Normal
Tracheal aspirate	Moraxella catarrhalis
Chest X-ray	Discrete perihilar infiltrates
Urine culture	Negative

**Table 3:** Investigations performed at the 5th hospitalisation, three years and eight months of age.

Investigation	Result
Blood smear	Atypical lymphocytes, no malignancy
Bone marrow aspiration	Hypoplastic marrow, no malignancy
Mutation analysis for FMF	Negative
ANA	Negative
ANCA	Negative
Anti-dsDNA	Negative
PCR parvovirus	Negative
PCR EBV	Negative
Lymphocyte subpopulations	Normal
Lymphocyte stimulation tests	Normal
IgD	2012.6 IU/mL (ref.: 11.4, 95% CI: 1–145.5)
Tuberculin skin test	Negative
Sweat test	Sweat Sodium 68 mmol/L (slightly elevated)
Delta 508 mutation analysis	Negative
X-ray thorax	Normal
Echocardiography	Small mitral insufficiency, not haemodynamic significant
US abdomen	Slight hepatomegaly with hyperechogenic patches
